# Simultaneous Correction of Juvenile Hallux Valgus and Flexible Flatfoot in Children: Outcomes of Combined First Metatarsal Hemiepiphysiodesis and Calcaneal-Stop Procedure

**DOI:** 10.3390/jcm14207330

**Published:** 2025-10-17

**Authors:** Giovanni Luigi Di Gennaro, Giovanni Trisolino, Marianna Viotto, Marco Todisco, Tosca Cerasoli, Gino Rocca

**Affiliations:** Pediatrics Orthopedics and Traumatology, IRCCS Istituto Ortopedico Rizzoli, 40136 Bologna, Italy; giovanniluigi.digennaro@ior.it (G.L.D.G.); marco.todisco@ior.it (M.T.); tosca.cerasoli@ior.it (T.C.); gino.rocca@ior.it (G.R.)

**Keywords:** first metatarsal hemiepiphysiodesis, LHFM, calcaneal-stop procedure, CS, combined CS

## Abstract

**Background/Objectives**: Juvenile hallux valgus (JHV) and flexible flatfoot (FFF) often coexist in children, yet their combined surgical management remains poorly explored. This study evaluates clinical and radiographic outcomes following a simultaneous approach using lateral hemiepiphysiodesis of the first metatarsal (LHFM) and calcaneal-stop (C-Stop) procedures in skeletally immature patients. **Methods**: A retrospective cohort of 24 bilateral patients (48 feet) aged 10–12 underwent LHFM and C-Stop between 2017 and 2023. Radiographic evaluation included Hallux Valgus Angle (HVA), Intermetatarsal Angle (IMA), Meary’s angle (MA), and transverse TaloCalcaneal (Kite’s) Angle (tTCA). The Foot and Ankle Disability Index (FADI) and the Tegner Activity Scale (TAS) were administered at the most recent follow-up and complications were recorded. **Results**: The mean follow-up was 3.7 years. Postoperative radiographs showed significant improvements in all parameters, with correction inversely correlated to baseline deformity severity. Full normalization of flatfoot parameters was achieved in 68.8% of feet, with mild residual deformity in the remainder. Males showed greater radiographic correction than females. IMA and HVA improved in most cases, reaching full normalization in 53.1% and 50% of feet, respectively. Clinically, all patients showed corrected hindfoot alignment and medial arch restoration; 90% achieved the maximum FADI score and 88% resumed recreational sports. Two cases of screw migration occurred, with one revision; no further complications were reported. **Conclusions**: Simultaneous correction of FFF and JHV using C-Stop and LHFM proved effective, yielding significant radiographic improvements and excellent functional outcomes in most cases, with minimal complications. However, full hallux alignment was achieved in only half of the cases, suggesting that additional distal metatarsal procedures may be needed for more severe deformities.

## 1. Introduction

Juvenile hallux valgus (JHV) and flexible flatfoot (FFF) are among the most prevalent foot deformities in the pediatric population and frequently present concomitantly [1]. The reported prevalence of JHV in children ranges from 2% to 4%, with notably higher rates observed among patients with flatfoot deformity [2]. Although traditionally managed as separate entities, these deformities often share underlying pathophysiological mechanisms, including ligamentous laxity, altered foot biomechanics, and abnormal joint alignment [3,4].

FFF is characterized by medial arch collapse, hindfoot valgus, and forefoot abduction during stance and gait. Although often asymptomatic, some children experience pain, fatigue, functional limitations during physical activity, difficulty with footwear, and psychological or aesthetic concerns [5,6]. JHV, on the other hand, is associated with genetic predisposition and generalized joint hypermobility and tends to worsen with growth, frequently resisting conservative treatment approaches [1,7].

Historically, surgical correction of JHV was postponed until skeletal maturity to avoid recurrence and growth plate injury [8]. However, guided growth techniques such as lateral hemiepiphysiodesis of the first metatarsal (LHFM) have emerged as promising alternatives, allowing gradual correction while preserving the growth potential of the first ray [9,10]. Similarly, subtalar arthroereisis techniques such as the calcaneal-stop (C-Stop) screw have demonstrated long-term efficacy, safety, and simplicity in treating symptomatic pediatric FFF by stabilizing the subtalar joint and restoring the medial longitudinal arch [11,12].

Despite increasing evidence supporting these techniques individually, their combined application in patients with coexisting JHV and FFF remains unreported. The biomechanical interdependence of the hindfoot and forefoot suggests that concurrent correction may enhance overall outcomes [13,14]. Previous studies have underscored this relationship, with authors advocating for comprehensive correction strategies in pediatric foot deformities [13,14,15].

This study aims to evaluate the clinical and radiographic outcomes of a combined surgical approach using LHFM and C-Stop in skeletally immature patients with JHV and FFF. We hypothesize that this dual-modality intervention will provide a synergistic effect in achieving functional and anatomical correction with minimal complications.

## 2. Materials and Methods

### 2.1. Study Design

We conducted a retrospective study of pediatric patients diagnosed with both FFF and JHV, treated at a single tertiary pediatric orthopedic center between 2017 and 2023. Inclusion criteria were idiopathic cases in skeletally immature patients over 10 years of age with open physes who underwent simultaneous treatment with C-Stop and LHFM. Exclusion criteria included rigid flatfoot, flatfoot associated with a short Achilles tendon, non-idiopathic cases, isolated procedures, incomplete clinical and radiographic documentation or lack of parental consent. Diagnosis was made by a single senior orthopedic surgeon with over 30 years of experience, based on clinical findings of FFF with a third- to fourth-degree plantar imprint (Viladot’s criteria) and/or hindfoot valgus > 10°, associated with symptomatic JHV, and characterized by medial deviation of the first metatarsal and lateral deviation of the big toe. Radiographic confirmation required an intermetatarsal angle > 9° and/or hallux valgus angle (HVA) > 15° combined with an abnormal Meary’s angle > 10° [16]. The study was approved by our ethics committee (Comitato Etico di Area Vasta Emilia Centro 32/2020/Oss/IOR approved on 6 February 2020), and informed consent was obtained from all patients and their guardians for participation.

### 2.2. Surgical Technique

The surgical technique for the calcaneal-stop (C-Stop) procedure has been previously described by the authors [17]. Briefly, the anterograde C-Stop was performed under local anesthesia with sedation and a tourniquet, with the patient in supine position, the leg slightly internally rotated, and the foot in maximal supination and neutral dorsiflexion. A 1–2 cm incision was made over the sinus tarsi. The lateral tubercle of the talus was identified and prepared using a straight awl directed perpendicularly toward the medial malleolus. Under fluoroscopic guidance, a custom-designed screw (Still Easyfoot, DialOrtho, San Pietro in Casale, Italy) was inserted at approximately 45° on the coronal plane and 0–35° posteriorly on the sagittal plane. The screw head remained anterolateral, effectively limiting pronation without joint intrusion. Final heel alignment was assessed with the knee extended in dorsiflexion, and screw position was confirmed intraoperatively using C-arm fluoroscopy. The titanium alloy screw features a broad, conical, smooth head that provides wide contact with the sinus tarsi, distributing loads evenly and minimizing the risk of loosening, penetration, or implant failure. The bottleneck junction between the head and threaded portion enhances stress distribution and reduces breakage risk, while the dual-pitch threading improves grip within the talus and prevents migration. In over 6400 implants, only 3 cases of screw breakage or cut-out were reported (0.05%, unpublished data from the authors).

**Figure 1:**
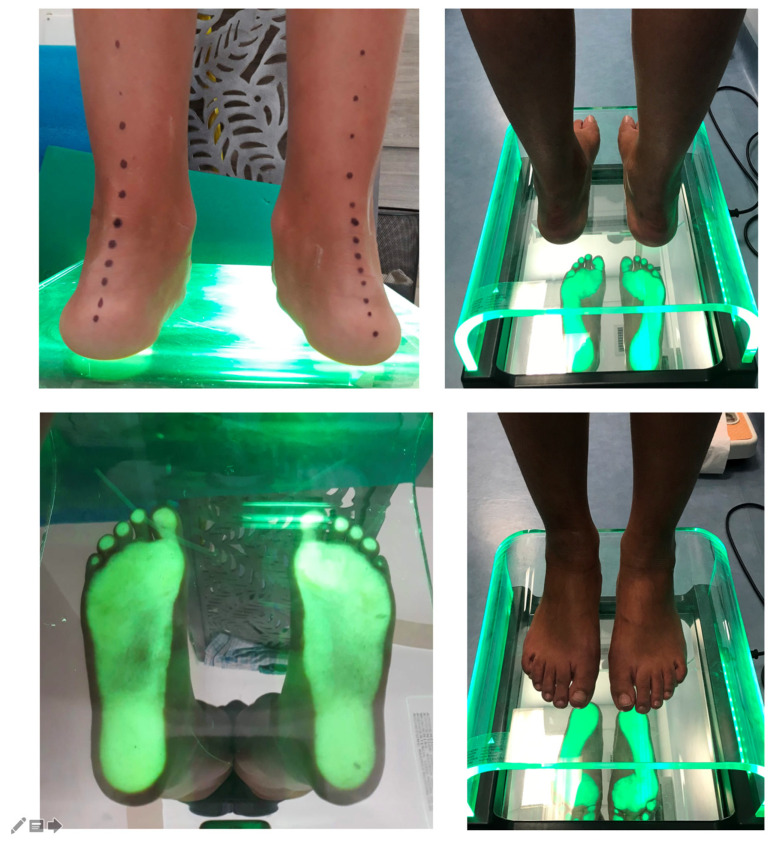
Preoperative clinical presentation and podoscopic evaluation of hallux valgus with flatfoot (**left**) and two-year postoperative clinical presentation and podoscopic evaluation (**right**).

**Figure 2:**
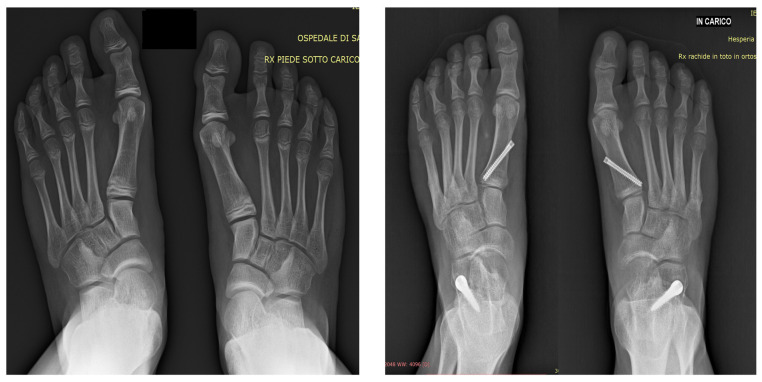
Standing preoperative radiographic appearance of flatfoot with hallux valgus (**left**) and standing radiographic appearance at two years postoperatively (**right**).

Once the calcaneal-stop screw is placed, the LHFM screw is inserted. A Mini Acutrak screw (Acumed LLC, Marmon Medical—Berkshire Hathaway Company Hillsbor, Oregon, US, is typically used (tip: 3.5 mm; tail: 3.6 mm; 2 mm increments; length 16–30 mm). Under fluoroscopic guidance, a guidewire is inserted from the medial cortex of the first metatarsal shaft toward the lateral aspect of the distal epiphysis, crossing the lateral third of the proximal physis. Screw length is determined using a dedicated gauge or by comparing with a second guidewire. The guidewire is then slightly advanced to avoid accidental displacement during drilling. A cannulated drill is used over the guidewire, and a fully threaded, headless, self-tapping screw is inserted to minimize hardware prominence and reduce the risk of irritation or soft tissue conflict. The clinical and radiological aspect of the patient before and after the surgery is depicted in Figure 1 and Figure 2.

### 2.3. Baseline and Outcome Variables

Pre- and postoperative data were collected and analyzed by independent observers who were not involved in the surgical decision-making process or in performing the procedures. Baseline data collected from medical records and radiographs included sex, age at surgery, whether screw removal was performed, and age at screw removal. Preoperative clinical symptoms (pain, fatigue, footwear difficulties) were collected from medical charts; however, a standardized pain VAS was not routinely recorded, representing a limitation of the present series. Two authors collected the following preoperative radiographic parameters: intermetatarsal angle (IMA), hallux valgus angle (HVA), Meary’s angle (MA), and transverse TaloCalcaneal Angle (tTCA). The IMA was defined as the angle between the longitudinal axes of the first and second metatarsals. Values < 9° were considered normal; 9–13° indicated mild, 13–20° moderate, and >20° severe hallux valgus [18]. The HVA, measured between the axes of the first metatarsal and the proximal phalanx, was considered normal below 15°; 15–30° indicated mild, 30–40° moderate, and >40° severe deformity [18]. MA, assessed on the lateral radiograph as the angle between the axes of the talus and first metatarsal, was used to evaluate medial arch collapse. Values of 0–4° were considered normal; 4–15° indicated mild, and values > 15° indicated moderate flatfoot [19]. tTCA was measured between the long axes of the talus and calcaneus on AP views. Normal values were 24 ± 6° [20], with higher values indicated increasing hindfoot valgus and flatfoot severity. Preoperative measurements were compared to postoperative values. A change of ≥1.85° in IMA [21] and ≥5.64° in HVA was defined a priori as the minimal clinically important difference (MCID) to identify meaningful progression or improvement. Complications, recurrence, and reoperations were also recorded.

At final follow-up, patients were re-contacted and the Italian cross-culturally adapted versions of Foot and Ankle Disability Index (FADI), a validated questionnaire assessing functional status in daily and sports activities, with scores ranging from 0 (maximum disability) to 100 (no disability) on the Tegner Activity Scale, were administered [22].

### 2.4. Statistical Analysis

Continuous variables are presented as mean ± standard deviation and range, while categorical variables are reported as counts and percentages with 95% confidence intervals. Data distribution was assessed for normality using the Kolmogorov–Smirnov test for continuous variables and exact tests for categorical variables. Non-parametric tests were applied when data did not meet normality assumptions. Within-group comparisons were performed using paired *t*-tests with Levene’s correction to account for unequal variances, or Wilcoxon signed-rank tests for non-normally distributed data. Associations between continuous variables (e.g., FADI scores, demographics, radiographic parameters) were analyzed using Pearson correlation coefficients or Spearman’s rho when normality was not met. Correlation strength was interpreted as follows: R < 0.3, negligible or very weak; 0.3 ≤ R < 0.5, weak; 0.5 ≤ R < 0.7, moderate; 0.7 ≤ R < 0.9, strong; and R ≥ 0.9, very strong [23]. A two-tailed *p*-value < 0.05 was considered statistically significant.

## 3. Results

A total of 24 patients (17 females, 7 males; 48 feet) were evaluated, with a mean age at surgery of 11.4 ± 0.7 years (range: 10.3–13.6) and a follow-up of 3.7 ± 0.9 years (range: 2.5–5.2). Females underwent surgery on average one year earlier than males, a statistically significant difference (*p* = 0.001). Preoperatively, 76.6% (IMA) and 89.1% (HVA) of feet were classified as having mild hallux valgus, while 23.4% (IMA) and 10.9% (HVA) were moderate; no severe cases were observed (the results are reported in Table 1). HVA was higher in females (mean difference: 5°; *p* = 0.010), with no sex-related differences in IMA. Based on MA, 27.8% of feet had mild and 72.2% moderate flatfoot, with no preoperative sex differences noted for flatfoot angles. We found a weak correlation between preoperative IMA and preoperative tTCA (R = 0.36; *p* = 0.038), as well as between preoperative HVA and preoperative Meary’s angle (R = 0.46; *p* = 0.010; see Supplementary Materials). Postoperative radiographs showed significant improvements across all parameters (Table 1), with a moderate to strong negative correlation (R = −0.5 to −0.8) between baseline deformity severity and degree of correction achieved (see Supplementary Materials). MA improved in all cases, with greater postoperative correction observed in males (mean difference 3.5° ± 1.2° compared to females). Full normalization was achieved in 68.8% of feet, while the remaining 31.2% showed residual mild flattening despite clear improvement. The talocalcaneal angle (tTCA) also decreased in all cases by an average of 6.7°, with a significantly greater reduction in males (−9.3°) compared to females (−5.5°; *p* = 0.010).

IMA significantly improved (≥MCID of 1.85°) in 63.3% of cases and fully normalized in 53.1%, with mild residual hallux valgus in the others. HVA normalized in 50% of cases; in the remaining half, mild deformity persisted. A significant reduction in HVA (≥MCID of 5.64°) was observed in 46.7%, a non-significant reduction in 40%, and mild, non-significant worsening in 10.3%. Clinically, all patients demonstrated improved hindfoot alignment with correction of calcaneal valgus and restoration of the medial arch, resulting in a type 1 plantar imprint according to Viladot. Functionally, the mean FADI score was 103 ± 2.5 (range: 96–104), with 90% of patients achieving the maximum score. The mean FADI Sport score was 31.5 ± 2 (range: 23–32). Both FADI and FADI Sport showed a weak inverse correlation with postoperative HVA (Spearman’s r = –0.41; *p* = 0.035). Within one year, 88% of patients returned to regular recreational sports, although none resumed competitive or professional-level activity. The mean TAS score was 3.5 ± 1.4 (range: 2–8), and showed a moderate positive correlation with improvement in MA (Spearman’s r = 0.54; *p* = 0.004). With the numbers available, we did not observe any further significant association between clinical outcomes and either sex, age at surgery, pre-operative and post-operative radiographic outcomes or screw removal timing. Two cases of screw migration occurred, requiring revision in one patient. No infections, neurovascular injuries, or overcorrections were observed. During the follow-up period, 10 patients (20 feet) underwent screw removal at a mean of 36 ± 11 months (range, 22–50), while the screws were left in place in the other patients, since they remained clinically asymptomatic.

## 4. Discussion

To our knowledge, this is the first study evaluating the combined use of C-Stop and LHFM for the simultaneous treatment of FFF and JHV. Our results show that this approach offers favorable clinical and radiographic outcomes with a low complication rate.

First and foremost, our findings strengthen the existing evidence on the safety and efficacy of the C-stop technique. As widely documented, subtalar arthroereisis, both endosinotarsal and exosinotarsal, consistently improves foot posture and radiographic parameters in pediatric FFF [5,24,25,26,27,28,29]. Our results are in line with several recent meta-analyses confirming the superior short- and long-term efficacy of the C-stop technique compared to other surgical options (particularly endorthesis and osteotomies) which, despite achieving similar radiographic correction, tend to yield less favorable functional outcomes [25,26,29]. Notably, our results align with those of Galán-Olleros et al., whose meta-analysis of 20 studies (2394 feet) reported mean improvements of 7.32° in tTCA and 11.65° in MA, closely matching those observed in our cohort [5].

In our series, although radiographic correction was significant, complete normalization was achieved in only ~70% of cases, with mild residual flattening persisting in the remaining 30%, predominantly among females. Incomplete radiographic correction has already been noted by Smolle et al. [25], who, in a long-term systematic review, observed that improvements in MA often remain within a range consistent with mild residual flatfoot, averaging 9° at over 48 months of follow-up. While all studies report significant angular improvements, the literature lacks data on the proportion of patients achieving full radiographic normalization. Most reports instead focus on clinical residual arch collapse, which has been described in 13–15% of cases [30,31].

Secondly, our results confirm the partial but consistent effectiveness of LHFM in correcting or at least limiting the progression of JHV associated with FFF. The mean correction of 5.9° for HVA and 2.6° for IMA aligns with values reported in several recent systematic reviews [7,9,14,32]. However, this limited corrective potential likely reflects minimal impact on the distal first metatarsal and the first metatarsophalangeal joint, resulting in only about half of the cases achieving full realignment of the first ray within normal parameters. With our sample size, no association was found between flatfoot correction and improvements in hallux valgus parameters, aligning with Mazzotti et al., who reported no significant hallux valgus correction after isolated subtalar arthroereisis [33].

Regarding JHV, we observed a predominance of mild deformities, with slightly higher angular values in females and only weak correlations with hindfoot radiographic parameters. Although FFF is slightly more common in males, our series showed a female predominance, likely due to the higher prevalence of hallux valgus in females [34]. This trend may also reflect a greater influence of aesthetic concerns on surgical decision-making, particularly among girls. While we did not collect preoperative patient-reported outcomes to confirm this, prior studies suggest that social perception and cosmetic concerns often influence the choice to undergo surgery for pediatric foot deformities [35].

Regarding the mild severity of cases in our cohort, it is worth noting that commonly used severity thresholds are based on adult populations, with no established pediatric-specific criteria. For instance, an HVA of 25° and an IMA of 12° may be classified as mild or moderate depending on the reference used [36,37]. Given the progressive nature of the deformity with age [38,39], it is plausible that the cut-offs for juvenile hallux valgus should be revised downward. Moreover, the weak correlation between hindfoot angles and hallux valgus has been debated by other studies, which similarly reported no to significant associations between hallux valgus angles and flatfoot parameters [1,6,40,41,42]. This supports the hypothesis of at least two distinct forms of JHV: a primary type, driven by intrinsic abnormalities of the first metatarsal (such as metatarsus primus varus, long first metatarsal and increased distal metatarsal articular angle, oblique first metatarsal–cuneiform joint, abnormal shape of the first metatarsophalangeal joint [34]), possibly explaining the presence of isolated cases of JHV even in the absence of FFF; and a secondary form, associated with excessive foot pronation, which tends to develop later and progress more gradually. However, longitudinal data to definitively confirm this distinction are still lacking.

Our findings can be better contextualized within the growing body of literature on juvenile hallux valgus. Recent systematic reviews and case series have highlighted that hemiepiphysiodesis of the first metatarsal can provide partial correction and slow progression of the deformity, although normalization of radiographic parameters is uncommon [7,10,14,43,44]. In contrast, minimally invasive distal osteotomies, such as SERI or MICA, achieve larger angular correction and significant functional improvement, but at the cost of greater surgical morbidity [32,45,46]. The recent comprehensive review by Seidenstein et al. confirmed that conservative management remains the first-line strategy in skeletally immature patients, while surgical intervention should be considered for persistent pain or progressive deformity, with hemiepiphysiodesis emerging as a promising but still under-investigated option [3]. Taken together, these data suggest that growth modulation may represent a low-morbidity alternative in selected pediatric patients, while osteotomies should be reserved for severe or refractory cases (a summary of the main studies on hemiepiphysiodesis and other surgical techniques for juvenile hallux valgus is provided in Supplementary Table S1).

Consistent with the previous literature, we observed that greater initial deformity was associated with a smaller degree of angular correction, consistent with the nature of this growth-guiding procedure, whose corrective potential is inherently limited and partly dependent on the child’s growth capacity and rate. Although in our series no significant correlation emerged between age at surgery and correction achieved, earlier intervention may offer better outcomes by extending the effective treatment window with implants in situ. Nonetheless, surgery before age 10–11 is generally discouraged due to the likelihood of spontaneous improvement, especially in flatfoot parameters. Overall, these findings highlight the need for caution and thorough counseling in cases of moderate to severe deformity, as the procedure—while safe and well tolerated—may prove insufficient and require additional interventions to achieve optimal results.

The role of LHFM remains debated, given the complex etiology of JHV and its often partial corrective effect, limited to the proximal metatarsal. The limited rate of complete correction observed in our series may be explained by the fact that several patients were treated close to skeletal maturity, when residual growth potential was insufficient to allow full remodeling. Earlier treatment might have favored higher correction rates. Nevertheless, even partial correction can be considered a valuable outcome, as it reduces deformity severity and facilitates any future corrective osteotomy starting from a less severe baseline. While LHFM may replace proximal metatarsal osteotomies, its impact on distal deformities appears limited. In such cases, distal metatarsal osteotomies (also via minimally invasive techniques) combined with bunionectomy, capsular procedures, or even distal medial hemiepiphysiodesis (while preserving epiphyseal blood supply to avoid avascular necrosis) may play a greater role [8,13,15,32,45,47]. A combined proximal and distal approach could be more effective in severe cases, although comparative studies in the pediatric population are still lacking.

From a functional perspective, nearly all patients resumed recreational sports within the first postoperative year, but none returned to competitive activity. This finding is consistent with long-term series of calcaneo-stop, where only a small minority of patients were engaged in high-impact competitive sports despite excellent functional scores [48]. The modest Tegner scores observed in our cohort (mean 3.5) reflect the typical profile of children with painful flexible flatfoot, who often present with overweight and generalized ligamentous laxity. These baseline characteristics are not representative of competitive athletes and likely explain why, although functional recovery was excellent, participation remained limited to recreational sports.

### Strengths and Limitations

The novelty of the combined treatment, the encouraging outcomes, and some noteworthy correlations represent key strengths of our study. However, several limitations must be acknowledged. The main limitation is the absence of a control group (e.g., C-Stop alone), which precludes isolating the incremental contribution of LHFM and limits causal inference. Moreover, the retrospective design inherently limits study quality, particularly due to the absence of preoperative patient-reported outcomes, which prevents a clear assessment of clinical improvement and its relationship with radiographic changes. The small sample size reduces statistical power and limits the ability to identify reliable predictors of treatment success or failure. Moreover, the lack of control groups, especially patients treated with C-Stop alone, makes it difficult to isolate the corrective effect of LHFM from that of hindfoot realignment. We treated only a few cases with isolated LHFM, who were excluded from this analysis; however, they may offer useful comparisons to better understand the additive or synergistic effects of simultaneous versus staged procedures. Future studies should compare hemiepiphysiodesis and osteotomy, both radiographically and functionally, as treatment options for mild JHV associated with FFF in the pediatric population.

## 5. Conclusions

In summary, the combined use of C-Stop and LHFM appears to be a safe and effective option for treating coexisting FFF and JHV in children, offering simultaneous correction with minimal morbidity and good functional outcomes. Radiographic correlations suggest a biomechanical link between hindfoot and forefoot alignment, supporting the rationale for a combined approach. However, full correction of hallux alignment was not achieved in all cases, indicating that additional distal procedures may be required in more severe deformities.

## Figures and Tables

**Table 1:** Pre- to postoperative changes in hallux valgus and foot angles. Values are reported as mean ± standard deviation. Mean differences were assessed using paired Student’s *t*-test and were all statistically significant (*p* < 0.0005). The abbreviations used are as follows: Intermetatarsal Angle (IMA), Hallux Valgus Angle (HVA), transverse TaloCalcaneal Angle (tTCA), Meary’s angle (MA).

Variable	Pre-Op	Post-Op	Mean-Difference (95% C.I.)
IMA (°)	11.9 ± 1.3	9.3 ± 1.5	2.6 ± 1.7 (2.0–3.2)
HVA (°)	22.0 ± 6.0	16.1 ± 3.6	5.9 ± 5.4 (3.8–7.9)
tTCA (°)	27.3 ± 3.3	20.6 ± 3.5	6.7 ± 3.6 (5.2–8.3)
MA (°)	18.8 ± 5.8	5.4 ± 4.8	13.3 ± 4.8 (11.4–15.3)

## Data Availability

Data are available upon reasonable request because of the privacy of patients.

## Supplementary Materials

The following supporting information can be downloaded at: https://www.mdpi.com/article/10.3390/jcm14207330/s1, Table S1: Summary of the recent studies reporting outcomes of hemiepiphysiodesis and minimally invasive surgical techniques for juvenile hallux valgus.

## Abbreviations

The following abbreviations are used in this manuscript:

LHFM	Lateral hemiepiphysiodesis of the first metatarsal
JHV	Juvenile hallux valgus
FFF	Flexible flatfoot
C-Stop	Calcaneal-stop
HVA	Hallux Valgus Angle
IMA	Intermetatarsal Angle
MA	Meary’s angle
tTCA	Transverse TaloCalcaneal Angle
FADI	Foot and Ankle Disability Index
TAS	Tegner Activity Scale

## References

[1] Kim H.W., Park K.B., Kwak Y.H., Jin S., Park H. (2019). Radiographic Assessment of Foot Alignment in Juvenile Hallux Valgus and Its Relationship to Flatfoot. Foot Ankle Int..

[2] Coughlin M.J., Jones C.P. (2007). Hallux Valgus: Demographics, Etiology, and Radiographic Assessment. Foot Ankle Int..

[3] Seidenstein A.H., Torrez T.W., Andrews N.A., Patch D.A., Conklin M.J., Shah A. (2022). Pediatric Hallux Valgus: An Overview of History, Examination, Conservative, and Surgical Management. Paediatr. Child Health.

[4] Wong D.W.-C., Wang Y., Chen T.L.-W., Yan F., Peng Y., Tan Q., Ni M., Leung A.K.-L., Zhang M. (2020). Finite Element Analysis of Generalized Ligament Laxity on the Deterioration of Hallux Valgus Deformity (Bunion). Front. Bioeng. Biotechnol..

[5] Galán-Olleros M., Del Baño Barragán L., Figueroa M.J., Prato de Lima C.H., Fraga-Collarte M., Torres-Izquierdo B., Hosseinzadeh P., Martínez-Caballero I. (2024). Outcomes of the “Calcaneo-Stop” Procedure for Treating Symptomatic Flexible Flatfoot in Children: A Systematic Review and Meta-Analysis of 2394 Feet. Foot Ankle Surg..

[6] Atbaşı Z., Erdem Y., Kose O., Demiralp B., Ilkbahar S., Tekin H.O. (2020). Relationship Between Hallux Valgus and Pes Planus: Real or Fiction?. J. Foot Ankle Surg..

[7] Al-Mohrej O.A., Ade-Conde A.M., Ade-Conde O.S., Argan M., Khan M., Bouchard M., Al-Asiri J. (2023). Hemiepiphysiodesis for Juvenile Hallux Valgus Deformity: A Systematic Review. Foot Ankle Surg..

[8] Hung W.-C., Yao S.-H., Wang T.-M., Chen C.-H. (2022). Transosseous Suturing for the Correction of Juvenile Hallux Valgus: A Preliminary Case Series Study. Medicina.

[9] AlZeedi M., Park J.P., Marwan Y., Abu-Dalu K.M., Hamdy R., Janelle C. (2023). Growth Modulation for the Treatment of Juvenile Hallux Valgus: A Systematic Review of Literature. Strateg. Trauma Limb Reconstr..

[10] Sabah Y., Rosello O., Clement J.L., Solla F., Chau E., Oborocianu I., Rampalv V. (2018). Lateral Hemiepiphysiodesis of the First Metatarsal for Juvenile Hallux Valgus. J. Orthop. Surg..

[11] Silva S., Tavernini T., Bruschi A., Andriolo L., Guizzardi G., Serra M., Rocca G., Filardo G. (2025). Calcaneo-Stop for Paediatric Idiopathic Flexible Flatfoot: High Functional Results and Return to Sport in 644 Feet at Mid-Term Follow-Up. J. Exp. Orthop..

[12] Stichnoth M., Lüders K.A., Hell A.K., Stinus H. (2025). Comparative Study of Subtalar Arthroereisis, Medializing Calcaneal Osteotomy and the Combination of Both Techniques for the Treatment of Symptomatic Adult Flatfeet. Foot Ankle Surg..

[13] Faldini C., Nanni M., Traina F., Fabbri D., Borghi R., Giannini S. (2016). Surgical Treatment of Hallux Valgus Associated with Flexible Flatfoot during Growing Age. Int. Orthop..

[14] Artioli E., Mazzotti A., Langone L., Zielli S.O., Arceri A., Bonelli S., Faldini C. (2023). First Metatarsal Hemiepiphysiodesis for the Treatment of Juvenile Hallux Valgus: A Systematic Review. J. Pediatr. Orthop..

[15] Yang F., Wu C., Wang J., Mei G., Zou J., Xue J., Su Y., Ma X., Zhang J., Shi Z. (2025). Subtalar Arthroereisis for Simultaneous Treatment of Flexible Pes Planus during Surgical Correction of Hallux Valgus. Eur. J. Med. Res..

[16] Polichetti C., Borruto M.I., Lauriero F., Caravelli S., Mosca M., Maccauro G., Greco T., Perisano C. (2023). Adult Acquired Flatfoot Deformity: A Narrative Review about Imaging Findings. Diagnostics.

[17] Di Gennaro G.L., Stallone S., Olivotto E., Zarantonello P., Magnani M., Tavernini T., Stilli S., Trisolino G. (2020). Operative versus Nonoperative Treatment in Children with Painful Rigid Flatfoot and Talocalcaneal Coalition. BMC Musculoskelet. Disord..

[18] Kuhn J., Alvi F. (2025). Hallux Valgus. StatPearls.

[19] Alsaidi F.A., Moria K.M. (2023). Flatfeet Severity-Level Detection Based on Alignment Measuring. Sensors.

[20] Meyr A.J., Wagoner M.R. (2015). Descriptive Quantitative Analysis of Rearfoot Alignment Radiographic Parameters. J. Foot Ankle Surg..

[21] Gong X.-F., Sun N., Li H., Li Y., Lai L.-P., Li W.-J., Wang Y., Wu Y. (2022). Modified Chevron Osteotomy with Distal Soft Tissue Release for Treating Moderate to Severe Hallux Valgus Deformity: A Minimal Clinical Important Difference Values Study. Orthop. Surg..

[22] Napoli R., Fortina M., Plebani G., Giannotti S., Pannone A., Rossi A., Visonà E., Tegner Y., Brindisino F., Vascellari A. (2023). Cross Cultural Adaptation and Multi Centric Validation of The Italian Version of The Tegner Activity Scale. Muscles Ligaments Tendons J..

[23] Moore D.S., Notz W.I., Fligner M.A. (2013). The Basic Practice of Statistics.

[24] Tan J.H.I., Tan S.H.S., Lim A.K.S., Hui J.H. (2021). The Outcomes of Subtalar Arthroereisis in Pes Planus: A Systemic Review and Meta-Analysis. Arch. Orthop. Trauma Surg..

[25] Smolle M.A., Svehlik M., Regvar K., Leithner A., Kraus T. (2022). Long-Term Clinical and Radiological Outcomes Following Surgical Treatment for Symptomatic Pediatric Flexible Flat Feet: A Systematic Review. Acta Orthop..

[26] De Pellegrin M., Moharamzadeh D. (2021). Subtalar Arthroereisis for Surgical Treatment of Flexible Flatfoot. Foot Ankle Clin..

[27] Sabry A.O., Genedy M.K.A., Hennidi M., Shebl M.A., Zaky A., Selim O.E.M., Shebl M.A., Hassan M.T.G., Almohani O., Arid M. (2024). Endosinotarsal vs. Exosinotarsal Subtalar Arthroereisis in Treating Pediatric Flexible Flat Feet: A Systematic Review and Meta-Analysis of Comparative Studies. JBJS Rev..

[28] Smith C., Zaidi R., Bhamra J., Bridgens A., Wek C., Kokkinakis M. (2021). Subtalar Arthroereisis for the Treatment of the Symptomatic Paediatric Flexible Pes Planus: A Systematic Review. EFORT Open Rev..

[29] Vescio A., Testa G., Amico M., Lizzio C., Sapienza M., Pavone P., Pavone V. (2021). Arthroereisis in Juvenile Flexible Flatfoot: Which Device Should We Implant? A Systematic Review of Literature Published in the Last 5 Years. World J. Orthop..

[30] Vogt B., Toporowski G., Gosheger G., Rölfing J.D., Rosenbaum D., Schiedel F., Laufer A., Kleine-Koenig M.-T., Theil C., Roedl R. (2021). Subtalar Arthroereisis for Flexible Flatfoot in Children-Clinical, Radiographic and Pedobarographic Outcome Comparing Three Different Methods. Children.

[31] Ali A., Walsh M., O’Brien T., Dimitrov B.D. (2014). The Importance of Submalleolar Deformity in Determining Leg Length Discrepancy. Surgeon.

[32] Lee S.W., Gabriel D., Lee D.W., Lin W.T.H., May C. (2025). Minimally Invasive Surgery for Juvenile Hallux Valgus. J. Pediatr. Orthop. Soc. N. Am..

[33] Mazzotti A., Langone L., Zielli S.O., Artioli E., Arceri A., Brognara L., Traina F., Faldini C. (2024). Do First Ray-Related Angles Change Following Subtalar Arthroereisis in Pediatric Patients? A Radiographic Study. Children.

[34] Chell J., Dhar S. (2014). Pediatric Hallux Valgus. Foot Ankle Clin..

[35] Dibello D., Dallan G., Di Carlo V., Pederiva F. (2023). Quality of Life in Flexible Painful Flatfoot Treated by Anterograde Calcaneo-Stop Procedure: The Patient’s and Family’s Perspective. PLoS ONE.

[36] Spindler F.T., Ettinger S., Baumbach S.F. (2024). Classification of Hallux Valgus Deformity-Is There a Standard?. Arch. Orthop. Trauma. Surg..

[37] Ray J.J., Friedmann A.J., Hanselman A.E., Vaida J., Dayton P.D., Hatch D.J., Smith B., Santrock R.D. (2019). Hallux Valgus. Foot Ankle Orthop..

[38] Scott G., Menz H.B., Newcombe L. (2007). Age-Related Differences in Foot Structure and Function. Gait Posture.

[39] Sung K.H., Kwon S.-S., Park M.S., Lee K.M., Ahn J., Lee S.Y. (2019). Natural Progression of Radiographic Indices in Juvenile Hallux Valgus Deformity. Foot Ankle Surg..

[40] McCluney J.G., Tinley P. (2006). Radiographic Measurements of Patients with Juvenile Hallux Valgus Compared with Age-Matched Controls: A Cohort Investigation. J. Foot Ankle Surg..

[41] Xie Y.L., Liang J.Y., Du G.F., Lu H.J., Luo W.J., Wu J.H., Sheng Z.H. (2025). The Causal Relationship between Hallux Valgus and Endogenous Pathogenic Factors: A 2-Sample Mendelian Randomization. Medicine.

[42] Jung D.-Y., Jung S.-H., Gwak G.-T. (2023). Contributions of Age, Gender, Body Mass Index, and Normalized Arch Height to Hallux Valgus: A Decision Tree Approach. BMC Musculoskelet. Disord..

[43] AlFarii H., Marwan Y., Algarni N., Addar A., Hamdy R., Janelle C. (2022). Temporary Screw Lateral Hemiepiphysiodesis of the First Metatarsal for Juvenile Hallux Valgus Deformity: A Case Series of 23 Feet. J. Foot Ankle Surg..

[44] Chiang M.H., Wang T.M., Kuo K.N., Huang S.C., Wu K.W. (2019). Management of Juvenile Hallux Valgus Deformity: The role of combined Hemiepiphysiodesis. BMC Musculoskelet Disord..

[45] Rocca G., De Venuto A., Mazzotti A., Zielli S.O., Artioli E., Brognara L., Traina F., Faldini C. (2023). The Minimally Invasive SERI Osteotomy for Pediatric Hallux Valgus. Children.

[46] Fontyn S., Alatassi R., Benaroch L.R., Pirshahid A.A., Bartley D., Carey T., Del Balso C., Thornley P. (2025). Treatment of Juvenile Hallux Valgus with a Simple, Effective, Rapid, and Inexpensive (SERI) Technique: A Systematic Review and Meta-Analysis. Foot.

[47] Knörr J., Soldado F., Violas P., Sánchez M., Doménech P., de Gauzy J.S. (2022). Treatment of Hallux Valgus in Children and Adolescents. Orthop. Traumatol. Surg. Res..

[48] Trisolino G., Ramella M., Pizzuti V., Todisco M., Parisi S.C., Cerasoli T., Rocca G. (2025). Ten-Year Clinical and Functional Outcomes of Anterograde Calcaneo-Stop Arthroereisis for Idiopathic Flexible Flatfoot in Children: A Single-Center Cohort Study. Children.

