# Supplementary material for: Simultaneous Correction of Juvenile Hallux Valgus and Flexible Flatfoot in Children: Outcomes of Combined First Metatarsal Hemiepiphysiodesis and Calcaneal-Stop Procedure
Source: J Clin Med. 2025 Oct 17;14(20):7330. doi: 10.3390/jcm14207330 (PMC12565386; doi:10.3390/jcm14207330)
Supplement: Supplementary file 1 [file jcm-14-07330-s001.zip › jcm-3832603-supplementary.pdf]

Journal of Clinical Medicine

MDPI

Table S1. Summary of the recent studies reporting outcomes of hemiepiphysiodesis and minimally invasive surgical techniques for juvenile hallux valgus.

Author (Year)	Study design / population	Mean Age	Technique	Key outcomes	Notes
Sabah et al. 2018 [10]	Prospective case series / 12 pts, 22 feet	10 y	Lateral hemiepiphysiodesis	Pain/function improved; partial angular correction	Safe, feasible, effect often partial
Al-Farii et al. 2022 [43]	Retrospective case series/ 14 pts, 23 feet	10.6 y	Temporary screw lateral hemiepiphysiodesis	Radiographic improvement, low complications	Technically simple; long-term data lacking
Artioli et al. 2023 [14]	Meta-analysis/ 85 pts, 144 feet	10.7 y	Hemiepiphysiodesis	Radiographic/clinical improvements; angles often remained mild/moderate	Hemiepiphysiodesis plays a bigger role in preventing the worsening of the deformity rather than correcting it
Al-Mohrej et al. 2023 [7]	Systematic review/ 85 pts, 147 feet	N.R.	Hemiepiphysiodesis	Confirms radiological and PROMs improvements	Safe, effective
Rocca et al. 2023 [45]	Retrospective case series / 45 pts, 58 feet	12.5 y	MIS SERI osteotomy	Confirm radiological and PROMs improvements, no major complications	MIS osteotomy gives greater angular correction
Fontyn et al. 2025 [46]	Meta-analysis/ 153 pts, 208 feet	14.6 y	SERI osteotomy	Significant radiographic and PROMs improvement	Confirms MIS safety and effectiveness
Chiang et al. 2019 [44]	Prospective case series/ 21 pts, 37 feet	12 y	Percutaneous hemiepiphysiodesis	Supports guided-growth rationale	Insufficient correction, minimally invasive, early evidence

pts = patients; y = years; N.R. = not reported; PROMs = patient-reported outcome measures; MIS = minimally invasive surgery; SERI = Simple, Effective, Rapid, Inexpensive osteotomy.
